# Design, Synthesis, and Evaluation of New Tripeptides as COX-2 Inhibitors

**DOI:** 10.1155/2013/606282

**Published:** 2013-02-26

**Authors:** Ermelinda Vernieri, Isabel Gomez-Monterrey, Ciro Milite, Paolo Grieco, Simona Musella, Alessia Bertamino, Ilaria Scognamiglio, Stefano Alcaro, Anna Artese, Francesco Ortuso, Ettore Novellino, Marina Sala, Pietro Campiglia

**Affiliations:** ^1^Dipartimento di Scienze Farmaceutiche e Biomediche, Università degli Studi di Salerno, Via Ponte don Melillo, 84084 Fisciano, Italy; ^2^Dipartimento di Chimica Farmaceutica e Tossicologica, Università degli Studi di Napoli Federico II, Via D. Montesano 49, 80131 Napoli, Italy; ^3^Dipartimento Farmaco-Biologico, Università degli Studi di Messina, Viale Annunziata, 98168 Messina, Italy; ^4^Dipartimento di Biochimica e Biofisica, Seconda Università degli Studi di Napoli, Via L. De Crecchio 7, 80138 Napoli, Italy; ^5^Dipartimento di Scienze della Salute, Università degli Studi “Magna Græcia” di Catanzaro, Campus Universitario “S. Venuta”, Viale Europa, 88100 Catanzaro, Italy

## Abstract

Cyclooxygenase (COX) is a key enzyme in the biosynthetic pathway leading to the formation of prostaglandins, which are mediators of inflammation. It exists mainly in two isoforms COX-1 and COX-2. The conventional nonsteroidal anti-inflammatory drugs (NSAIDs) have gastrointestinal side effects because they inhibit both isoforms. Recent data demonstrate that the overexpression of these enzymes, and in particular of cyclooxygenases-2, promotes multiple events involved in tumorigenesis; in addition, numerous studies show that the inhibition of cyclooxygenases-2 can delay or prevent certain forms of cancer. Agents that inhibit COX-2 while sparing COX-1 represent a new attractive therapeutic development and offer a new perspective for a further use of COX-2 inhibitors. The present study extends the evaluation of the COX activity to all 20^3^ possible natural tripeptide sequences following a rational approach consisting in molecular modeling, synthesis, and biological tests. Based on data obtained from virtual screening, only those peptides with better profile of affinity have been selected and classified into two groups called S and E. Our results suggest that these novel compounds may have potential as structural templates for the design and subsequent development of the new selective COX-2 inhibitors drugs.

## 1. Introduction

The main cause of the inflammation is the prostaglandins overproduction, which are synthesized by cyclooxygenase enzymes [1].

**Figure 1:**
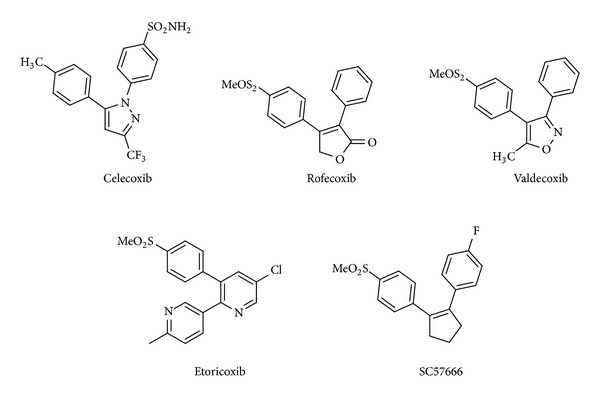
Chemical structures of selective COX-2 inhibitors.

Prostaglandin-endoperoxide synthase, commonly called cyclooxygenase (COX), is an intracellular enzyme required for the conversion of arachidonic acid to prostaglandins. The two best-known COX isoforms are referred to as COX-1 and COX-2 for the order in which they were discovered [2]. The first isozyme is constitutively expressed in resting cells of most tissues, functions as a housekeeping enzyme, and is responsible for maintaining homeostasis (gastric and renal integrity) and normal production of prostaglandins; *vice versa*, the COX-2 expression is induced by infection and it is responsible for the inflammatory response. Such a difference suggested to report COX-1 as the constitutive form and COX-2 as the inducible one. More recently, the constitutive presence of COX-2 has been highlighted in brain, kidney, and endothelial cells but is virtually absent in most other tissues. In particular, COX-2 expression is significantly upregulated as part of various acute and chronic inflammatory conditions and in neoplastic tissues. The design of a selective inhibitor is difficult as the COX-1 and COX-2 binding sites are almost identical, and the isoforms show sequence homology of 60–65%. Experimental 3D models of both enzyme structures have shown the complexity of the problem. They suggest that the tertiary conformations of these proteins are very similar, and the substrate binding pocket and catalytic site amino acids are nearly identical in both enzymes. In this case, the only difference is the substitution, at residue position 523, of the COX-1 Ile by the COX-2 Val that, opening an additional pocket, results in an enzyme inducible form binding site larger than the constitutive one. The structural similarities of COX-1 and COX-2 enzymes have made the development of selective inhibitors for COX-2 versus COX-1 a special challenge. Since the discovery of the COX-2 enzyme in the early 1990s, numerous COX-2 selective inhibitors have been proposed (Figure 1).

A common structural feature of these selective COX-2 inhibitors is the presence of two vicinal aryl rings attached to a central five- or six-membered heterocyclic or carbocyclic motif. Typical examples of selective COX-2 inhibitors like celecoxib, rofecoxib, valdecoxib, etoricoxib, and SC57666 demonstrate that a broad variety of five- or six-membered carbo- and heterocycles are acceptable for binding to the cyclooxygenase active site.

Recent reviews on the current status of COX-2 inhibitors further confirm the flexibility of the carbocyclic/heterocyclic core motif for COX-2 binding [3]. Good results were obtained with the coxib family, but their secondary effects, especially affecting the kidney and central nervous system, stimulated the research towards the most powerful substances with a lower isoform selectivity. On the basis of the previously reported information, the present study extends the evaluation of the COX activity to all 20^3^ possible natural tripeptide sequences following a rational approach consisting in molecular modeling, synthesis, and biological tests.

## 2. Materials and Methods

### 2.1. Molecular Modeling

The PDB entries 1Q4G [4] and 1PXX [5] have been considered as COX-1 and COX-2 receptor models, respectively. The crystallographic resolution, equal to 2.0 Å for 1Q4G and to 2.9 Å for 1PXX, and the kind of cocrystallized ligands, two reversible NSAIDs, have been the choice criterion. The original ligands, *α*-methyl-4-biphenylacetic acid and diclofenac, respectively, have been removed, and hydrogen atoms, missing into the PDB files, have been added by means of the Maestro GUI [6]. In order to appropriately take into account structural solvent, all cocrystallized water molecules, within 5 Å from the protein atoms, have been included in our preliminary models. Added hydrogen atoms position has been optimized according to MMFFs [7–10] force field implemented in MacroModel ver. 7.2 [11]. The procedure consisted in a preliminary energy minimization computed applying to all nonhydrogen atoms a constant force equal to 200 KJ/mol·Å followed by a further water molecules unconstrained run. Using the same force field and the same constraints, both receptor models final structures have been submitted to 500 ps of molecular dynamics at 300°K, with an integration time step equal to 1.5 fs. In all simulation a distance-dependent dielectric constant equals to 4 has been adopted. Such an approach highlighted the most tightly interacting water molecules that maintained their position, while the other ones have been moved far from the receptor models. In order to achieve a fully relaxed conformation, resulting molecular dynamics structures, containing the tightly interacting solvent molecules only, have been submitted to unconstrained energy minimization producing our COX-1 and COX-2 receptor models.

The 3D tripeptides library was built by an *in-house* PerlMol Chemistry modules-based software. All possible combination of 20 the (*L*)-series natural amino acids have been considered, and the resulting 8000 tripeptide models were energy minimized using the same force field reported for the receptors.

The docking binding site, for both targets, has been defined by means of a regular box centered onto the protein chain A Tyr 385. Its volume was about 390,000 Å^3^ widely covering COX-1 and -2 binding sites. Glide grid maps have been computed using the standard precision algorithm. Our optimized library has been submitted to flexible Glide docking simulation with respect to both COX receptor models reducing the ligands van der Waals atom radius by a 0.8 factor. Default Glide interaction energies (ECvdW) have been adopted for scoring the tripeptide docking poses.

### 2.2. Materials

*N* ^*α*^-Fmoc-protected amino acids, Wang resin, HOBt, HBTU, DIEA, piperidine, and trifluoroacetic acid were purchased from Iris Biotech (Germany). Peptide synthesis solvents, reagents, and CH_3_CN for HPLC were reagent grade and were acquired from commercial sources (Sigma Aldrich, Italy) and used without further purification unless otherwise noted.

### 2.3. Synthesis

The synthesis of tripeptides (S1–E10) was performed according to the solid phase approach using standard Fmoc methodology in a manual reaction vessel [12]. The first amino acid, N^*α*^Fmoc-Xaa-OH, was linked onto the Wang resin (100–200 mesh, 1% DVB,1.1 mmol/g) and was attached to Wang resin using HOBt/HBTU as an activating agent (3eq.) and a catalytic amount of DMAP.

The following protected amino acids were then added stepwise: N^*α*^-Fmoc-Met-OH, N^*α*^-Fmoc-Arg(Pbf)-OH, N^*α*^-Fmoc-His(*N*_(*im*)_trityl (Trt))-OH (or N^*α*^-Fmoc-Gln(Trt)-OH) N^*α*^-Fmoc-Gly-OH, N^*α*^-Fmoc-Trp(Boc)-OH, N^*α*^-Fmoc-Glu(OtBu-OH, N^*α*^-Fmoc-Asp(OtBu)-OH, N^*α*^-Fmoc-Phe-OH, N^*α*^-Fmoc-Lys(Boc)-OH, N^*α*^-Fmoc-Ala-OH, N^*α*^-Fmoc-Ser(tBu)-OH, and N^*α*^-Fmoc-Ile-OH. Each coupling reaction was accomplished using a 3-fold excess of amino acid with HBTU and HOBt in the presence of DIEA (6 eq.). The N^*α*^-Fmoc protecting groups were removed by treating the protected peptide resin with a 25% solution of piperidine in DMF (1 × 5 min and 1 × 25 min). The peptide resin was washed three times with DMF, and the next coupling step was initiated in a stepwise manner. The peptide resin was washed with DCM (3×), DMF (3×), and DCM (3×), and the deprotection protocol was repeated after each coupling step.

In addition, after each step of deprotection and after each coupling step, Kaiser test was performed to confirm the completeremoval of the Fmoc protecting group, respectively, and to verify that complete coupling has occurred on all the free amines on the resin.

The N-terminal Fmoc group was removed as described above, and the peptide was released from the resin with TFA/iPr_3_SiH/H_2_O (90 : 5 : 5) for 3 h. The resin was removed by filtration, and the crude peptide was recovered by precipitation with cold anhydrous ethyl ether to give a white powder and then lyophilized.

### 2.4. Purification and Characterization

All crude peptides were purified by RP-HPLC on a semipreparative C18-bonded silica column (Phenomenex, Jupiter, 250 × 10 mm) using a Shimadzu SPD 10A UV/VIS detector, with detection at 215 and 254 nm.

The column was perfused at a flow rate of 3 mL/min with solvent A (10%, v/v, water in 0.1% aqueous TFA), and a linear gradient from 10 to 90% of solvent B (80%, v/v, acetonitrile in 0.1% aqueous TFA) over 40 min was adopted for peptide elution. Analytical purity and retention time (tR) of each peptide were determined using HPLC conditions in the above solvent system (solvents A and B) programmed at a flow rate of 1 mL/min using a linear gradient from 10 to 90% B over 25 min, fitted with C-18 column Phenomenex, Jupiter C-18 column (250 × 4.60 mm; 5 *μ*).

All analogues showed >97% purity when monitored at 215 nm. Homogeneous fractions, as established using analytical HPLC, were pooled and lyophilized.

Peptides molecular weights were determined by ESI mass spectrometry. ESI-MS analysis in positive ion mode, were made using a Finnigan LCQ ion trap instrument, manufactured by Thermo Finnigan (San Jose, CA, USA), equipped with the Excalibur software for processing the data acquired. The sample was dissolved in a mixture of water and methanol (50/50) and injected directly into the electrospray source, using a syringe pump, which maintains constant flow at 5 *μ*L/min. The temperature of the capillary was set at 220°C (Table 1).

### 2.5. Biological Assay

#### 2.5.1. Preparation of Washed Platelets

 Washed human platelets were prepared from blood anticoagulated with citrate-phosphate-dextrose, which was obtained from Centro de Transfusion de Galicia (Santiago de Compostela, Spain).

Bags containing buffy coat from individual donors were diluted with the same volume of washing buffer (NaCl, 120 mM; KCl, 5 mM; trisodium citrate, 12 mM; glucose, 10 mM; sucrose, 12.5 mM; pH 6) and centrifuged at 400 g for 9 min. The upper layer containing platelets (platelet-rich plasma) was removed and centrifuged at 1000 g for 18 min. The resulting platelet pellet was recovered, resuspended with washing buffer, and centrifuged again at 1000 g for 15 min. Finally, the platelet pellet from this step was resuspended in a modified Tyrode-HEPES buffer (HEPES 10 mM; NaCl 140 mM; KCl 3 mM; MgCl_2_ 0.5 mM; NaHCO_3_ 5 mM; glucose 10 mM; pH 7.4) to afford a cell density of 3–3.5·10^8^ platelet/mL. The calcium concentration in the extracellular medium was 2 mM [13].

#### 2.5.2. hCOX Activity

 The potential effects of the test drugs on total hCOX activity (bis-dioxygenase and peroxidase reactions) were investigated by measuring their effects on the oxidation of N,N,N′,N′-tetramethyl-p-phenylenediamine (TMPD) to N,N,N′,N′-tetramethyl-p-phenylenedaimine, using AA as common substrate for both hCOX-1 and hCOX-2, microsomal COX-2 prepared from insect cells (Sf21 cells) infected with recombinant baculovirus containing cDNA inserts for hCOX-2 (Sigma Aldrich Química S.A., Alcobendas, Spain), and COX-1 from human platelet microsomes (obtained as described in the above paragraph since, unlike hCOX-2, hCOX-1 is not available commercially) [14].

## 3. Results and Discussion

### 3.1. Design

The purpose of this work consists in the identification of new peptide ligands for COX that show, compared to the current state of the art, a greater power, a lower toxicity, and a high average degree of selectivity. 

In particular, the attention has been focused on cyclooxygenase 2 (COX-2) as it has been recently shown to promote multiple events in the tumorigenesis process [15]. Several reports indicate that COXs inhibitors can prevent the development of various human tumors including colon, breast, lung, liver, and gastric neoplasias [16–20]. 

For several types of cancer, the real risk factor seems to be chronic inflammation that maintains a high level of COX-2 and increases events that promote tumor formation. A tragic example of this mechanism is malignant mesothelioma (MM), a rare tumor of the mesothelial surface of the pleural and peritoneal cavities [21].

The aim of this study is to demonstrate the possibility of modulation of the activity of COX through peptides that may be found in sites in which the inflammatory process is in place.

To achieve this goal, we relied on the data reported in the literature, focused the attention on the structure of COX-2. One of the known potent inhibitors of COX-2 is SC-558 (a dyaril heterocyclic inhibitor) which contains a bromophenyl ring, a pyrazole group, and a phenylsulphonamide moiety. Most of the sulphur containing NSAIDs are selective COX-2 inhibitors, and this sulphur atom reduces the toxicity of the compound.

Using this information, in a recent work, Somvanshi et al. have designed and synthesized several tripeptide sequences containing a hydrophobic amino acid with aromatic ring, a cysteine residue which contains sulphur atom, and a charged residue at the C-terminal end [1]. In particular, 15 tripeptides were screened by ELISA test and the best of them, tripeptide WCS, inhibited more than 85% of the COX-2 activity.

Though the chemical nature of sulphur atom of sulphonamide group in SC-558 and in cysteine residue is different, still similarities in binding constant of peptide with known NSAIDs SC-558 were observed. Thus, preventing the reaction of substrate arachidonic acid with the enzyme supports the possibility of peptide WCS as potent and competitive inhibitor of COX-2. As the phenyl ring of SC-558 interacts with residues in hydrophobic cavity of COX-2 formed by Phe 381, Leu 384, Tyr 385, Trp 387, Phe 518, and Ser 530, it can be assumed that the aromatic ring of tryptophan residue of peptide will also interact with residues in hydrophobic cavity. The free carboxylate group of the peptide can electrostatically interact to Arg 120. WCS can be considered as a potential *lead compound* for developing a new class of COX-2 inhibitors. 

Extending the study of Somvanshi et al. [1], we have built a complete tripeptide virtual library containing all possible combinations of the 20 (*L*)-series natural amino acids. The COX-1 and -2 binding pocket recognition, of the 8000 library hits, has been investigated by means of molecular docking techniques, and the resulting complexes stability has been evaluated using the theoretical ligand-enzyme binding energies. The selection of the peptides, for the experimental tests, has been driven by their affinity with respect to both COX isoforms. Such a task has been carried out by computing the ratio between COX-2 and COX-1 peptide interaction energy. The top two COX-2 interacting compounds, still maintaining affinity for the COX-1, and one theoretically inactive peptide have been selected. The first couple corresponds to the sequences Gly-Met-Asp (GMD) and Gly-His-Glu (GHE), while the last one is Tyr-Tyr-Val (YYV). GMD and GHE showed a strong recognition to COX-2 and a weaker interaction to COX-1; YYV was unable to fit into both binding pockets due to its steric hindrance (Table 2).

**Figure 2:**
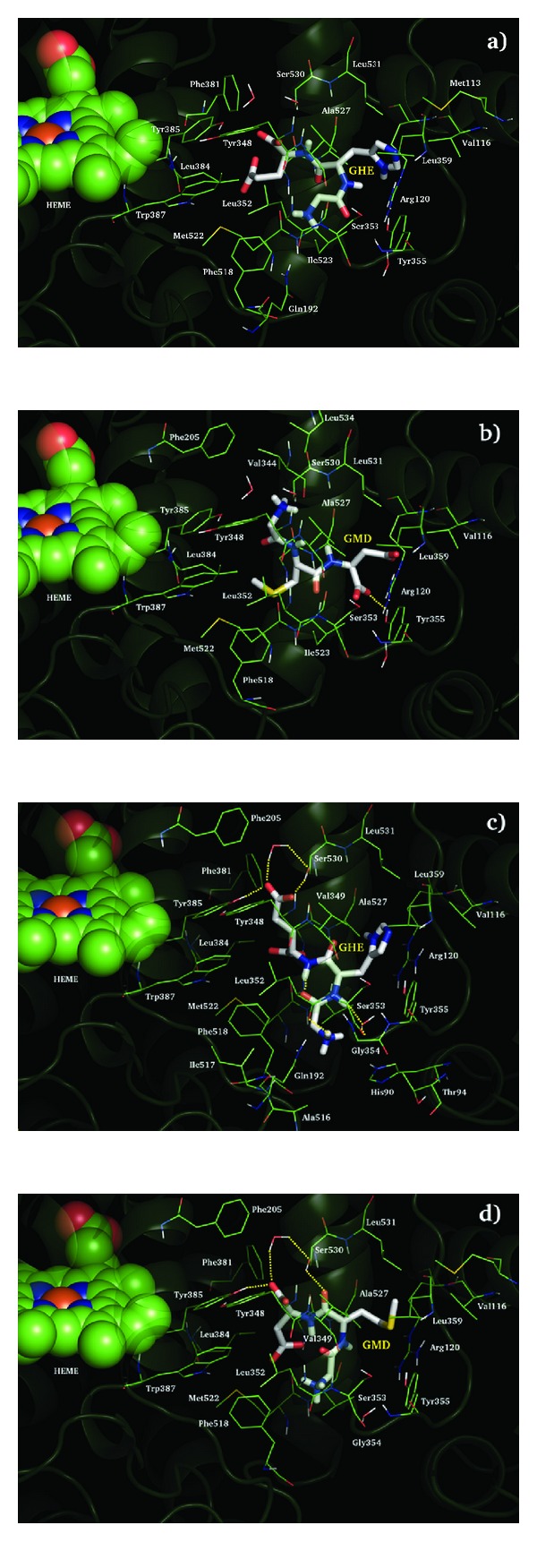
COX-1 (a) and (b) and COX-2 (c) and (d) recognition of GMD and GHE. Tripeptides are depicted in polytube CPK colored notation, interacting enzyme residues in green carbons wireframes, HEME cofactor in green carbons spacefill, and the rest of the enzyme is showed in transparent green cartoon. Yellow dotted lines indicated hydrogen bond interaction.

The graphical inspection of the GMD and GHE COXs complexes revealed, for both peptides, remarkable different recognition of the two enzymes (Figure 2).

In all cases our compounds have occupied the known NSAID binding pocket. Interestingly, the number of enzyme interacting residues, comprise between 21 and 26, is quite similar, but the COX-2 hydrogen bond network is much better than COX-1 and could explain the selectivity of our ligands. COX-2 Ser 530 side chain is involved in hydrogen bond to GMD backbone and, through a water molecule bridge, to the carboxylate groups of both our active peptides. Tripeptide carboxylate moieties are, also, involved in hydrogen bonds to COX-2 catalytic Tyr 385. GHE still reports such an interaction between its protonated N-terminal and Leu 352 backbone. Even if GMD, through its C-terminal, shows one hydrogen bond to Tyr 355 and favorable electrostatic interaction to COX-1 Arg 120, the steric hindrance of COX-1 Ile 523, bulkier than COX-2 Val, limits the enzyme cleft recognition preventing our compounds, in particular GHE, from establishing hydrogen bonds highlighted in COX-2. The remaining part of contribution to the COXs recognition of our peptides could be addressed an unspecific van der Waals interaction. 

At the same time, based on data obtained from virtual screening, only those peptides with better profile of affinity have been selected and classified into two groups called S and E (Table 1). The first group includes 10 sequences capable of interacting with both enzymatic isoform, but with a clear preference towards COX-2. In the second group are placed further 10 tripeptides, which have shown affinity exclusively for COX-2 and no reconnaissance towards COX-1.

### 3.2. Chemistry

 All peptides S1-E10 were synthesized by standard 9-fluorenylmethoxycarbonyl (Fmoc) chemistry using an appropriate orthogonal protection strategy. Peptides were released from the solid support using a cleavage cocktail of 90% TFA, 5% water, and 5% Et_3_SiH. All analogues showed >97% purity at HPLC analysis.

### 3.3. Anti-infiammatory Activity

 The biological assays were carried out to evaluate the inhibitory activity against COX-2 by the group of Professor Dr. Francisco Orallo, Department of Pharmacology, Faculty of Pharmacy, University of Santiago de Compostela, Spain.

Results shown in Table 3 are expressed as means ± SEM from five experiments. Means were compared by one-way analysis of variance (ANOVA) followed by Dunnett's post-hoc test. The inhibitory effects of the tested compounds are expressed as IC_50_ (concentrations that produce reduction of 50% of the enzymatic activity of COX control isoform) estimated by least-squares linear regression using the program Origin 5.0, with *X *= log molar concentration of the tested compounds and Y = % of pharmacological response.

This regression was performed using the data obtained with 4–6 different concentrations of each compound assayed, which inhibited the enzymatic activity of COX control isoform between 20 and 80%.

Finally, they were calculated the corresponding indices of selectivity (SI) of COX-1. SI = [IC_50_(COX-2)]/[IC_50_(COX-1)].

As demonstrated by the virtual screening, all twenty tripeptides show a greater selectivity against COX-2 over COX-1.

In particular, peptide S9 shows a very interesting profile of both selectivity and inhibitory potency towards COX-2; in fact, the selectivity index between COX-2 and COX-1 is about 0.308, more selective than the nimesulide that has an index of about 0.46; moreover, this peptide also shows an increase in activity compared to the same drug (68.34 ± 5.43 *μ*M S9 activity, 231.40 ± 19.84 *μ*M nimesulide activity). 

Analyzing biological data, depending both on the chemical structure that the values of the energies of binding, the peptides S9, S10, S7 and S4 show an analogous biological profile (selectivity and affinity).

However, a complete analysis of the structure-activity relationship of these peptides cannot be performed because of the small number of peptides that limit the goodness of this report. It is possible to highlight two important aspects: the guanidine group of Arg at C-terminal and the carboxyl group in the side chain of the second amino acid are requirements for the interaction with the target, while all peptides that have a carboxyl group in the side chain on the first amino acid show a loss of selectivity that of affinity; the aromatic group present in WCS, peptide lead, is not essential to interact with the target.

In conclusion, previously reported peptides seem to reflect too high potency and selectivity; instead, peptides of series E do not result selective for COX-2 (data not shown). Further studies for the peptides E1–E10 are in progress. 

## 4. Conclusion

There is an increasing interest in the development of new treatments based on cyclooxygenases-2 inhibitors, to prolong survival and even potentially cure various forms of cancer, as malignant mesothelioma. 

The present study describes hit identification, synthesis, and biological evaluation of a series of linear tripeptides, most of them are able to selectively inhibit COX-2. Further, other experiments aimed to verify the potentiality of these peptides as anticarcinogenic drugs; as well as the preparation of novel; more potent and selective peptidomimetic derivatives are in progress. 

Abbreviations used for amino acids follow the rules of the IUPAC-IUB Commission of Biochemical Nomenclature in J. Biol. Chem. 1972, 247, 977-983. Amino acid symbols denote L-configuration.

## Figures and Tables

**Table 1:** Structures and analytical data of tripeptides synthesized.

Peptide	Structure	HPLC	ESI-MS		Yield
		tR	found	calc	
S1	GMD	10.50	322.08	321.10	75%
S2	ERA	9.99	375.3	374.19	80 %
S3	GHE	8.08	342.14	341.13	67%
S4	GER	10.00	361.17	360.18	80%
S5	DRC	9.89	393.20	392.15	62%
S6	ARA	10.46	317.24	316.19	68%
S7	PER	8.87	401.40	400.21	81%
S8	KHI	10.98	397.11	396.25	80%
S9	AER	11.00	375.32	374.19	75%
S10	AGR	9.03	303.34	302.17	79%
E1	SRH	8.95	399.30	398.20	68%
E2	SWE	8.04	421.0	420.16	69%
E3	IRT	8.03	389.3	388.24	76%
E4	SMD	8.00	342.16	351.11	78%
E5	GRN	8.43	346.2	345.18	65%
E6	SHE	8.68	372.17	371.14	76%
E7	SQE	8.45	363.17	362.14	67%
E8	SMH	9.46	374.26	373.14	75%
E9	ARM	8.03	377.6	376.19	77%
E10	AQE	9.97	347.0	346.15	76%

tR: peptide retention time.

**Table 2:** COXs theoretical binding energies (be) in kcal/mol, number of van der Waals interacting enzyme residues (ir), and intermolecular hydrogen bonds (hb).

Peptide	COX-1			COX-2		
	be	ir	hb	be	ir	hb
GMD	−1.20	23	1	−39.87	21	3
GHE	−0.56	25	0	−42.15	26	5
YYV	—	—	—	—	—	—

**Table 3:** Inhibitory activity of compounds synthesized and selectivity against COX-2 over COX-1.

	COX-1 (IC_50_)	COX-2 (IC_50_)	Ratio
Indometacina	12.16 ± 1.16 *μ*M	35.20 ± 1.41 *μ*M	2.9
Diclofenac	18.23 ± 1.73 *μ*M	23.62 ± 1.97 *μ*M	1.3
FR122047	93.80 ± 6.55 *μ*M	***	>1.066^a^
Nimesulide	***	231.40 ± 19.84 *μ*M	<0.46^a^
DuP697	22.61 ± 1.56 *μ*M	126.32 ± 7.41 *μ*M	0.0056
S1	150.33 ± 2.34 *μ*M	94.04 ± 2.59 *μ*M	0.6255
S2	143.21 ± 2.57 *μ*M	120.92 ± 2.33 *μ*M	0.8443
S3	152.44 ± 5.18 *μ*M	94.89 ± 2.12 *μ*M	0.6225
S4	99.32 ± 1.14 *μ*M	80.56 ± 2.14 *μ*M	0.8111
S5	161.43 ± 2.57 *μ*M	100.01 ± 2.33 *μ*M	0.6195
S6	102.31 ± 1.14 *μ*M	91.20 ± 2.41 *μ*M	0.8914
S7	100.33 ± 2.19 *μ*M	88.21 ± 3.01 *μ*M	0.8792
S8	122.48 ± 3.78 *μ*M	91.66 ± 2.98 *μ*M	0.7484
S9	221.57 ± 1.04 *μ*M	68.34 ± 5.43 *μ*M	0.308
S10	99.11 ± 1.55 *μ*M	79.20 ± 2.15 *μ*M	0.7991
E1–E10**	—	—	—

Significant differences between the two means (*P* < 0.05 or *P* < 0.01) were determined by one-way analysis of variability (ANOVA) followed by Dunnett's post hoc test.

***No active at 500 *μ*M (the highest concentration tested).

^a^Value obtained whereas the corresponding IC_50_ to COX-1 or COX-2 is the highest concentration tested.

**Data not shown.

## Abbreviations

DMAP: 4-dimethylamino-pyridine
TFA: Trifluoroacetic acid
DCM: Dichloromethane
DIPEA: N,N-diisopropylethylamine
DMF: N,N-dimethylformamide
Et_3_SiH: Triethylsilane
Fmoc: 9-fluorenyl-methoxycarbonyl
HOBt: N-hydroxy-benzotriazole
HBTU: 2-(1H-benzotriazole-1-yl)-1,1,3,3-tetramethyluronium hexafluoro-phosphate
RP-HPLC: Reversed-phase high performance liquid chromatography
ESI: Electrospray ionization
LC-MS: Liquid chromatography-mass spectrometry
Pbf: 2,2,4,6,7-pentamethyldihydrobenzofuran-5-sulfonyl.
